# Effects of Shoe Midsole Hardness on Lower Extremity Biomechanics during Jump Rope in Healthy Males

**DOI:** 10.3390/healthcare9101394

**Published:** 2021-10-18

**Authors:** Hai-Bin Yu, Wei-Hsun Tai, Jing Li, Rui Zhang, Wei-Ya Hao, Jian-Zhi Lin

**Affiliations:** 1 School of Physical Education, Quanzhou Normal University, Quanzhou 362000, China; yhb@qztc.edu.cn (H.-B.Y.); dlove520@hotmail.com (W.-H.T.); zhangrui@jlu.edu.cn (R.Z.); haoweiya@ciss.cn (W.-Y.H.); 2 College of Textiles and Apparel, Quanzhou Normal University, Quanzhou 362000, China; lijingzzq@163.com; 3 Key Laboratory of Bionic Engineering (Ministry of Education, China), Jilin University, Changchun 130022, China; 4 China Institute of Sport Science, General Administration of Sport of China, Beijing 100061, China; 5 Department of Physical Education, National Taiwan University of Sport, Taichung 40404, Taiwan

**Keywords:** footwear, biomechanics, stiffness

## Abstract

This study investigated differences in lower extremity muscle activations and vertical stiffness during a 2.2 Hz jump rope exercise with different midsole hardnesses (45, 50, 55, and 60 Shores C). Twelve healthy male participants wore customized shoes with different hardness midsoles and performed jump rope exercises in a random order. A nine-camera motion analysis system (150 Hz), a force platform (1500 Hz), and a wireless electromyography (EMG) system (Noraxon, 1500 Hz) were used to measure the biomechanical parameters during the jump rope exercise. The biceps femoris %MVC of barefoot participants was significantly greater than that of those wearing the 45 Shores C (*p* = 0.048) and 55 Shores C (*p* = 0.009) midsole 100 ms before landing. The vastus medialis %MVC of barefoot participants was significantly greater than that of those wearing the 55 C midsole (*p* = 0.005). Nonsignificant differences in vertical stiffness were found between midsole hardnesses and barefoot. Lower extremity muscle activation differed between conditions. The results of this study indicate that for repetitive activities that entail multiple impacts, sports shoes with a low midsole hardness (e.g., 50 Shores C or 45 Shores C) may be appropriate. It is important to provide customers with information regarding midsole hardness in shoe product labeling so that they properly consider the function of the shoes.

## 1. Introduction

Jump rope is an activity widely used in physical education and sport training to promote health and fitness [1]. Previous studies indicated that jump rope can be easily learned without training, and only a small space is needed [2,3]. Moreover, it is beneficial to cardiorespiratory [4], strength [5], agility [2], coordination [6], and bone health [7]. Jump rope requires the use of hands to rotate the rope under the foot and above the head while simultaneously performing a continuous hop jump with the legs [2]. According to the 2019 National Health Standards for Physical Fitness of Student from the Ministry of Education of China, a jump rope test is conducted as an element of entering higher education admission, which means that at least 282 million students must participate in this activity. Therefore, the potential risks of jump rope should be identified, and, as such, footwear selection is an important factor.

There are a considerable number of differences between being barefoot and wearing shoes; however, the functions of shoes can differ in relation to the differences in midsole characteristics, such as hardness, materials, and structure, which affect the sports performance and injury risk of the lower extremity musculoskeletal system [8,9,10]. Previous studies report that muscle activation adjusts the joint stiffness during the landing phase of repetitive impacts, such as jump rope [3,4,5,6], which was based on prior exercise experience and motor reflexes of muscles. Muscle activation is typically measured using electromyography (EMG), which reflects the changes in motor units in muscles, thereby showing the information regarding muscle function and activities [11]. When muscle activation is high, a large EMG amplitude increases, which provides an information of muscle function and control during motion analysis [12,13].

Previous research indicated the muscle activation is affected by hardness of shoe insoles [14]. Assessing average and peak muscle amplitudes is a common method in surface EMG measurement, and such assessment is also applied to the investigation of muscle activation, making comparisons between barefoot participants and those [8]; however, to our knowledge, no studies have shown the systematic effects of midsole hardness on lower limb muscles. Footwear is highly correlated with lower extremity injury during exercise [15]. Appropriate footwear is expected to provide fine shock reduction and avoid stress injuries [16]. Harder midsole increases the impact force and plantar pressure which also increases the potential risk of injuries. Footwear that cannot reduce excessive and lower extremity repetition shock might increase injury risk [17,18,19]. Softer midsole hardness has been associated with better comfort and lower plantar pressure during simple movement tasks such as walking [20,21]. Several studies showed no significant difference in vertical stiffness during jump rope [22,23,24]. However, the function of midsole hardness is still not clear. Therefore, studying the changes in muscle activation while wearing customized shoes with different midsole hardnesses during jump rope exercise appears to be valuable.

The purpose of the present study was to examine the effect of midsole hardness on lower extremity muscle activation during jump rope exercise. It was hypothesized that lower muscle activation would be observed in shoes with a softer midsole.

## 2. Materials and Methods

### 2.1. Participants

Twelve healthy male university physical education students (age, 20.7 ± 3.4 years, stature, 170.7 ± 4.4 cm, body mass, 68.0 ± 3.8 kg) were recruited. The number of participants for each group was calculated with pilot data using G*Power software version 3.1.9.7 (ES = 0.61, α = 0.01, and 1-β = 0.95). The target foot length of participants was European size 41 and with dominant a right leg. Participants were asked with ability to jump rope at least 140/min and all of the, were free of any lower extremity injuries for 6 months prior to the start of the test. Before the experiment, all the participants read and signed an informed consent form. The study was conducted according to the guidelines of the Declaration of Helsinki and approved by the Ethics Committee of university of Taipei (UT IRB NO.: IRB-2016-021).

### 2.2. Instruments

The foot measurement was carried out using a YETI 3D foot scanner (Vorum Canada Inc., Vancouver, BC, Canada) to select potential participants who shoe size was European 41 and without any pes cavus (high arch) or flat foot (low arch). Kinematic and kinetic data were collected using nine infrared cameras (Raptor-E, Motion Analysis Corporation, Santa Rosa, CA, USA) at a 150 Hz sampling rate and a force platform (60 × 90 cm AMTI BP600900-6-2000; Advance Mechanical Technology, INC., Watertown, MA, USA) at a 1500 Hz sampling rate. The kinematic and kinetic data were synchronized and analyzed using Cortex software (Cortex 3.0, Motion Analysis Corporation, Rohnert Park, CA, USA).

The sports shoes were customized and the only difference of shoes was the thickness of midsole, whereby according to the hardness test (Shore C) the larger the Shore C number, the harder the midsole. Muscle activations were recorded using a wireless surface electrode (Noraxon USA Inc., Scottsdale, AZ, USA) which was placed on the muscles of the vastus medialis (VM), biceps femoris (BF), tibialis anterior (TA), and gastrocnemius muscle (GA) of the dominant leg at a 1500 Hz sampling rate. EMG signals were corrected through a band-pass filter at 20–400 Hz and full-wave rectified; the EMG data were normalized with the maximal voluntary isometric contraction (MVC) of each muscle (%MVC). The MVC was determined before the jump rope experiments, which was captured from the middle three seconds of the integral EMG. The EMG value of each stage divided by the time of the period to derive the average EMG value [9]. The ensemble peak EMG from the 3 MVC trials was used for normalized trials. Four EMG stages were divided by landings during jump rope cycles [10,11,12]. A Noraxon TeleMyo Direct Transmission System (DTS; Noraxon USA Inc., Scottsdale, AZ, USA) and the MR-XP1.07 Master Edition software package were employed and synced periodically with kinematic and kinetic data.

### 2.3. Procedures

**Figure 1:**
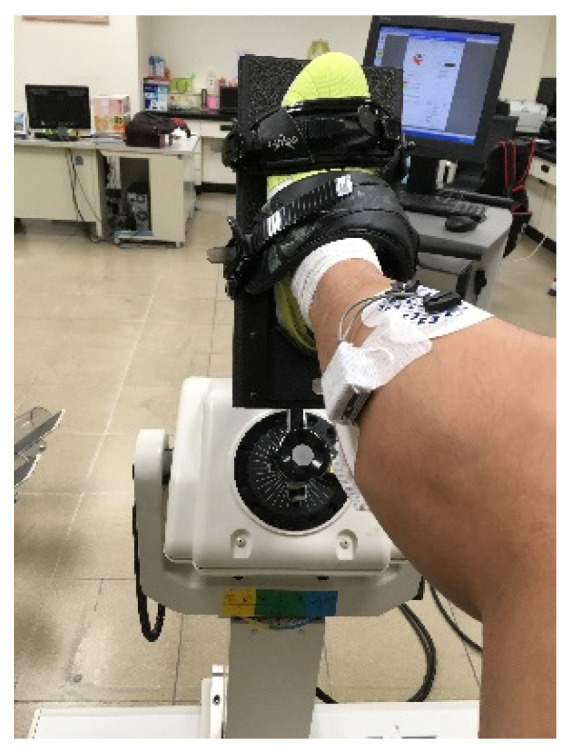
Biodex isokinetic dynamometer.

Participants started by engaging in 5–10 min dynamic warmup exercises. Prior to testing, the skin was shaved and swabbed with alcohol before a single-use electrode piece was attached to decrease EMG artifacts. The Biodex isokinetic system dynamometer (Medical Systems, Shirley, NY, USA), was used for MVC tested. Participants’ had their calf supported in padding and their foot secured in a foot plate (Figure 1). Each participant then had tests on four muscles (vastus medialis, biceps femoris, tibialis anterior, and gastrocnemius) to which they provided maximum voluntary isometric contractions by initiating a contraction and holding it at maximal effort for 5 s to allow for normalization of EMG signals.

**Figure 2:**
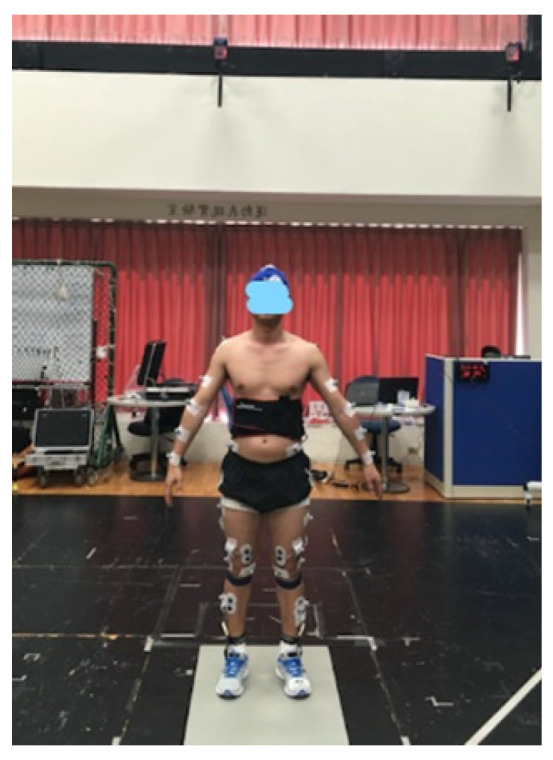
Experimental setup.

Subsequently, the jump rope test was conducted (Figure 2 displays the test setup). To avoid learning or fatigue effects in participants during the test, they were randomly assigned to perform the test barefoot or in sports shoes with Shore hardness testes of either 60, 55, 50, or 45 Shore C. The participants performed 2.2 Hz jump rope (single-under) [25] exercises for 30 s to ensure uniform jumping during tests with both feet on the force platform [26]; the speed of the jump rope was controlled by a metronome with BPM 130. Five trials of each jump rope exercise were carried out for each participant, and participants rested for 1 min between trials.

### 2.4. Data Reduction and Analysis

Body center of mass (COM) was defined by 29 retroreflective markers (19 mm in diameter) according to the modified Helen–Hayes configuration (Table 1). Vertical stiffness was calculated as the change in vertical ground reaction force divided by the change in vertical COM displacement [27]. The EMG analysis were referred to Hobara et al. [28,29]. The average muscle activity for 100 ms prior to landing was defined as the pre-activation (PRE) stage, and the average muscle activity 30 ms after landing was defined as the background activity (BGA) stage. The average muscle activity 30–60 ms after landing was defined as the supraspinal voluntary command to activate muscles and a short latency stretch reflex component (M1), and that 60–90 ms after landing was defined as the long latency stretch reflex component (M2).

### 2.5. Statistical Analysis

Statistical analysis was performed using SPSS 21.0 for Windows (IBM Corp., Armonk, NY, USA). Descriptive data expressed as the means (M) and standard deviations (SD) of the variables. The Shapiro–Wilk test was used to assess data normality. One-way repeated measures analysis of variance was performed to determine the effect of shoe hardness during jump rope exercise. A Bonferroni correction was used to adjust the *p*-value for multiple comparisons in tests, the alpha level for the statistical tests was set at α = 0.01 after Bonferroni correction adjust. The effect size (*η*^2^) for the differences was calculated to indicate the practical relevance of the significance.

## 3. Results

**Figure 3:**
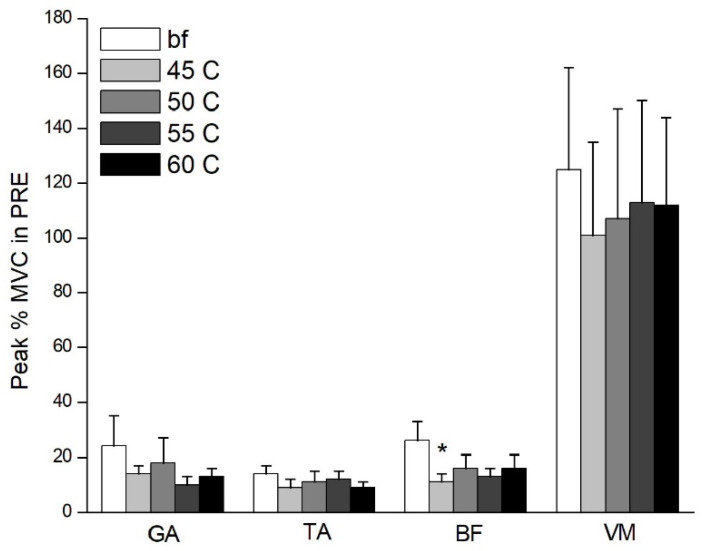
Peak %MVC of lower extremity in PRE stage. bf = barefoot. * Indicates significant difference with barefoot (*p* < 0.01).

**Figure 4:**
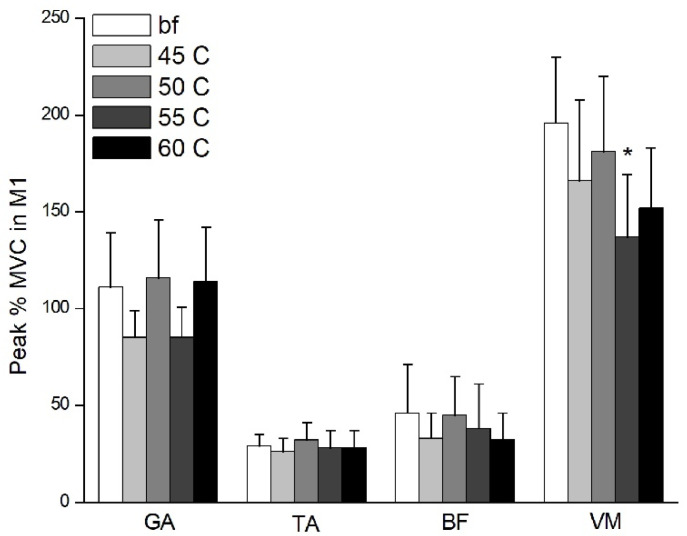
Peak %MVC of lower extremity in M1 stage. bf = barefoot. * Indicates significant difference with barefoot (*p* < 0.01).

The results show that the peak %MVCs of BF and VM muscles were significantly greater in barefoot participants than in those wearing shoes (Figure 3 and Figure 4). Bonferroni post hoc analysis shows that the BF of barefoot was significantly greater than that of midsole hardness of 45 C (*p* = 0.009, *η*^2^ = 0.284) in the PRE stage; VM of barefoot was significantly greater than that of midsole hardness of 55 C (*p* = 0.005, *η*^2^ = 0.224) in the M1 stage (Figure 4). No significant difference was found in the peak EMG of the TA, GA, or VM between barefoot or different hardness midsoles in the BGA stage (0.234 ≤ *p* ≤ 0.765, 0.041 ≤ *η*^2^ ≤ 0.142) and M2 stage (0.259 ≤ *p* ≤ 0.742, 0.043 ≤ *η*^2^ ≤ 0.118).

**Figure 5:**
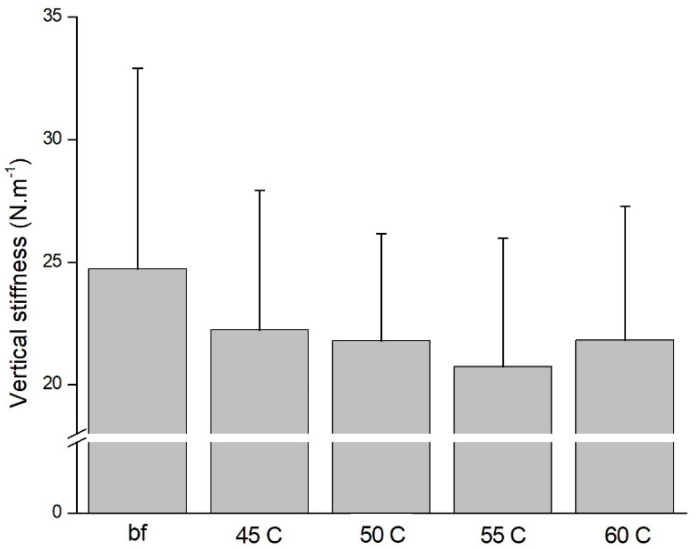
Vertical stiffness in different midsole hardness during jump rope exercise.

No significant difference was found in the average EMG of the TA, GA, BF, or VM between barefoot or different hardness midsoles in the four stages (0.093 ≤ *p* ≤ 0.859, 0.037 ≤ *η*^2^ ≤ 0.141). No significant difference was observed in vertical stiffness (*p* = 0.513, *η*^2^ = 0.072) during jump rope exercise among barefoot participants and those wearing shoes with midsole of different hardnesses (Figure 5).

## 4. Discussion

The purpose of the present study was to estimate the lower extremity muscle activation during consecutive, single jump rope exercise at 2.2 Hz and to compare these measurements in relation to midsole hardnesses. The results demonstrate that the biceps femoris of barefoot participants has a significantly greater peak %MVC than that of those wearing 45 Shores C and 55 Shores C midsole footwear during the PRE stage. Additionally, the vastus medialis of barefoot participants has a significantly greater peak %MVC than that of those wearing 55 Shores C midsole footwear during the M1 stage. This indicates that being barefoot may lead to higher muscle activation in order to maintain the stability of single jump rope exercise, which needs future research to prove. Although there were no significant differences in vertical stiffness, this study reaffirmed that appropriate footwear should be considered when participating in jump rope. Because of the shock-absorption design of sport shoes [18,19], they are an efficient means of injury prevention.

Compared with the EMG results of barefoot participants, it appears that wearing footwear may have reduced unnecessary muscle work during jump rope exercise. Nigg et al. [17] suggested that the hardness condition of the soles affects the amount of stress on joints and tendons because the muscle activation of the tibialis anterior and gastrocnemius increased under a harder midsole condition to maintain ankle stability during running. Notably, there were no differences found between midsole hardnesses. A previous study [26] reports on an impact test with different velocities and two midsole hardness conditions, and the results show that muscle activations did not change with shoe differences. There could be two reasons for such results: first, the small differences in midsoles and, second, the individual differences in jump rope skill.

The present results indicate that there was no difference in vertical stiffness among the various midsole hardnesses during the 2.2 Hz jump rope exercise. These results are consistent with those of previous research. Yu et al. [22] investigated the effects of different sports shoes on vertical stiffness among 20 participants performing jump rope exercises at 2.2 Hz for 30 s; no significant difference in vertical stiffness among the different shoes was observed. Moreover, Moncahi et al. [23] compared different vertical and leg stiffness between dominant and nondominant feet during 2.2 Hz jump rope exercise; no significant difference was found between feet. However, Divert et al. [30] found that vertical stiffness decreased among participants wearing shoes when running at 3.61 m/s when compared to that of those who were barefoot. Furthermore, stiffness, such as vertical stiffness and that in the joints and legs, is an important factor in adjusting the load balance of the human body [27]. It is important to consider footwear function when jumping rope; the various forms of lower extremity stiffness increased with longer hopping duration increase, which means the repetition impact might increase lower extremity injury risk [19]. An appropriate shoe may be helpful in offsetting the reductions in stiffness or external force that allow for the total movement to be maintained in all conditions. Bishop et al. [31] compared vertical stiffness in different functional sport shoes in continuous hop jumps of 2.2 Hz; no significant difference was found. However, Hobara et al. [28] investigated hop jumps at three different frequencies—1.5, 2.1, and 3.0 Hz—and they found that vertical stiffness increased with an increase in frequency. The present result showed a similar pattern in vertical stiffness, yet no significant difference was found. This is likely because the jump rope task and small range of midsole hardness control were not sufficient for a substantial effect on vertical stiffness. It must be borne in mind that jump rope exercise at 2.2 Hz is of considerably different intensity to that of running or fast hopping, and it is possible that the changes in midsole hardness were not perceivable by participants or occurred as a result of individual skill differences. Although vertical stiffness showed no difference in the present research, the minor damage occurring during jump rope exercise may increase the risk of lower extremity injury when performed without appropriate footwear [18].

There are some limitations to this study. First, we did not consider personal comfort of the shoes, and the EMG sensors could be affected the biomechanics parameters during jumping. Second, jump rope skills are influenced by sports and personal, individual differences. Finally, we only measured vertical stiffness and muscle activations during jump rope exercise with different midsole hardnesses. To better understand the influence of midsole hardness, future investigations should consider synchronized kinematics and kinetic measurements with different jump rope frequencies.

## 5. Conclusions

The present study investigated the effects of different hardness midsoles on lower extremity muscle activations and vertical stiffness during jump rope exercise. Higher muscle activation during jump rope exercise was observed for barefoot participants than for those wearing shoes. The levels of hardness of the midsoles showed minor differences during jump rope exercise, but these may have been concealed by individual skill differences. It is important to provide customers with information regarding midsole hardness in shoe product labeling so that they properly consider the function of the shoes. We suggest that a soft midsole is appropriate for repetitive sports with repeated impact.

## Figures and Tables

**Table 1:** The locations of retroreflective marker set.

No	Location	No	Location
1	Top. Head	16	R. Shank
2	Front. Head	17	R. Ankle
3	Rear. Head	18	R. Heel
4	R. Shoulder	19	R. Toe
5	R. Offset	20	L. Thigh
6	R. Elbow	21	L. Knee
7	R. Wrist	22	L. Shank
8	L. Shoulder	23	L. Ankle
9	L. Elbow	24	L. Heel
10	L. Wrist	25	L. Toe
11	R. ASIS	26	R. knee. Medial
12	L. ASIS	27	R. Ankle. Medial
13	V. Sacrum	28	L. knee. Medial
14	R. Thigh	29	L. Ankle. Medial
15	R. Knee		
